# Micronutrient availability alters *Candida albicans* growth and farnesol accumulation: implications for studies using RPMI-1640

**DOI:** 10.1128/spectrum.01571-24

**Published:** 2024-09-24

**Authors:** Cory H. T. Boone, Daniel J. Gutzmann, Jaxon J. Kramer, Shyanne D. Urbin, Dhammika H. Navarathna, Audrey L. Atkin, Kenneth W. Nickerson

**Affiliations:** 1 School of Biological Sciences, University of Nebraska, Lincoln, Nebraska, USA; 2 Central Texas Veterans Health Care System, Temple, Texas, USA; Stony Brook University, Stony Brook, New York, USA

**Keywords:** *Candida albicans*, farnesol, RPMI-1640, MOPS contamination, manganese, zinc, aromatic fusel alcohols, antifungal susceptibility testing

## Abstract

Science is challenging because we do not know what we do not know. Commercial chemicals are often marketed with >99% purity, but 0.5–1% impurity can impact results and cloud data interpretation. We recently developed an assay for farnesol and aromatic fusel alcohols from *Candida albicans*. During proof-of-concept experiments using RPMI-1640 growth media, the buffering compound was switched from MOPS obtained from Acros Organics to MOPS obtained from Sigma-Aldrich, both labeled 99% + purity. We observed a twofold decrease in growth, along with a three- to fivefold increase in farnesol production per cell upon the switch. ICP-MS showed that trace Mn(II) was present in Acros MOPS but absent in Sigma MOPS. Optimal growth was achieved by the addition of Mn(II), Zn(II), and Fe(II). We established upper and lower limits for Fe(II), Zn(II), Cu(II), and Mn(II) that allowed similar growth and then assessed 16 different mineral combinations in RPMI-1640 base media. The results show an increased production of farnesol and the aromatic fusel alcohols when Zn(II) is abundant, and a further increase in the aromatic fusel alcohols when both Fe(II) and Zn(II) are abundant. Finally, antifungal susceptibility testing displayed no significant difference between RPMI/MOPS with and without mineral supplementation. Supplemental Mn(II) was most needed for cell growth, while supplemental Zn(II) was most needed for the production of farnesol and the aromatic fusel alcohols. To avoid these artifacts due to metal contamination, we now use a modified RPMI supplemented with 1 mg/ L of Cu(II), Zn(II), Mn(II), and Fe(II).

**IMPORTANCE:**

The dimorphic fungus *Candida albicans* is a major opportunistic pathogen of humans. RPMI-1640 is a chemically defined growth medium commonly used with *C. albicans*. We identified over 32,000 publications with keywords RPMI and *C. albicans*. Additionally, Antifungal Susceptibility Testing (AFST) protocols in the United States (CLSI) and Europe (EUCAST) utilize RPMI as a base media to assess drug efficacy against clinical fungal isolates. RPMI contains many nutrients but no added trace metals. We found that the growth characteristics with RPMI were dependent on which MOPS buffer was chosen and the contamination of that buffer by trace levels of Mn(II) and Zn(II). Added Mn(II) was most needed for cell growth while added Zn(II) was most needed for secretion of farnesol and other signaling molecules.

## INTRODUCTION

**Fig 1:**
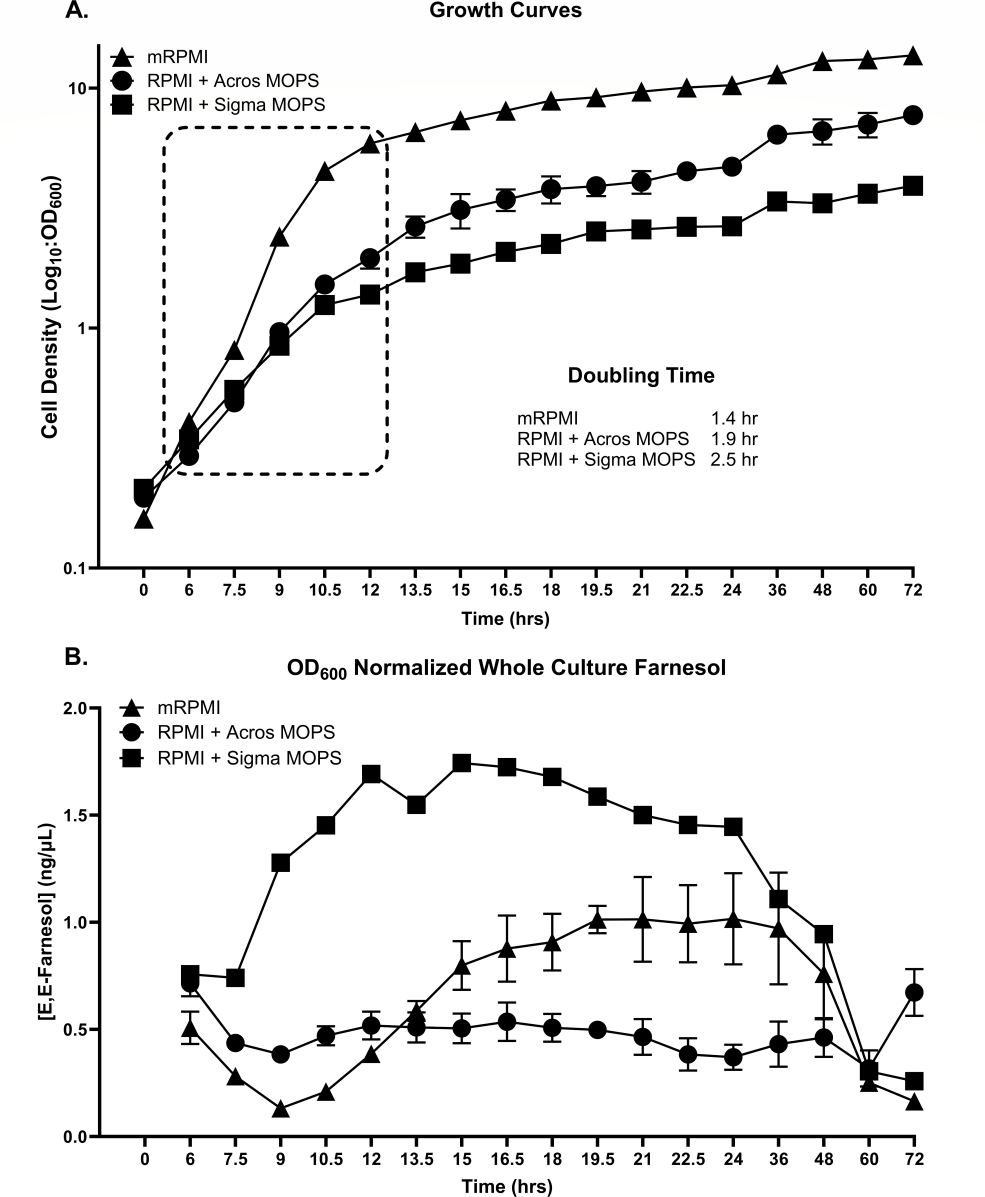
Aerobic growth curves and farnesol production over 72 h of growth for *C. albicans* in RPMI with different MOPS or trace metals. Cells were grown at 30°C with shaking at 225 rpm. (**A**) Growth curves in mRPMI, triangles (*n* = 3); RPMI + Acros MOPS, circles (*n* = 2); and RPMI + Sigma MOPS, squares (*n* = 1). Doubling times were calculated over the 6-to-12-h time frame, highlighted by the dashed box. Doubling time curves fit *R*^2^ values of 0.9857 (mRPMI), 0.9784 (Acros), and 0.9987 (Sigma). (**B**) Cell density normalized whole culture farnesol production for mRPMI, triangles (*n* = 3); RPMI + Acros MOPS, circles (*n* = 2), and RPMI + Sigma MOPS, squares (*n* = 1). The mRPMI data in Fig. 1B are the same as previously reported (2).

The importance of RPMI as a fungal growth medium is clearly shown by a Dimensions search with keywords of RPMI and *Candida albicans* which identified 32,396 publications (1). The dimorphic fungus *C. albicans* is a major commensal and opportunistic fungal pathogen of humans. It secretes farnesol as both a signaling molecule and a virulence factor (1). Recently, as a necessary step in defining the physiological functions of farnesol, we described an improved method for quantifying and distinguishing whole culture, cell-associated, and supernatant farnesol levels (2). As a proof of principle experiment to validate this method, we measured farnesol production in triplicate for 60 h on two of the commonly used growth media for *C. albicans*: YPD (yeast extract-peptone-dextrose) and RPMI-1640. RPMI is a chemically defined growth medium containing glucose, 20 amino acids, 11 vitamins, and five or six inorganic salts, but no added trace metals. As expected, growth and farnesol production for the YPD grown replicates were very tightly clustered (2). However, when we measured growth and farnesol levels for the RPMI grown cells (Fig. 1), we found that the third RPMI replicate was wildly disparate (Fig. 1B). For the purposes of our methods paper, we supplemented RPMI with a mineral mix to provide final concentrations of 1 mg/L of Cu(II), Zn(II), Mn(II), and Fe(II) salts so that the proof of principle data for our improved assay used modified RPMI (mRPMI) grown cultures exclusively (2).

The present paper expands on these previous results with two purposes in mind. The first is to alert the fungal community to an insidious pitfall concerning MOPS. The cell growth and farnesol production differences observed among presumed replicates (Fig. 1) are due to varying levels of trace metal contamination introduced by different batches of MOPS used in formulating RPMI/MOPS. The healthcare system gold standard protocol in both the United States (CLSI) and Europe (EUCAST) utilizes RPMI/MOPS for antifungal susceptibility testing (AFST) (3). Many academic labs, including ours, use these media when studying pathogenic fungi because it is the gold standard. We now report different experimental results when using MOPS from different manufacturers. Our second purpose takes advantage of RPMI being metal deficient to establish a baseline for the physiological roles of the respective trace metals. Supplemental Mn(II) was most needed for cell growth while supplemental Zn(II) was most needed for production of farnesol and the aromatic fusel alcohols.

## RESULTS

### Influence of MOPS and trace metals on cell growth

This line of research stems from our previous methods paper (2), where we found that the batch of MOPS used to prepare RPMI/MOPS strongly influenced cell yields and farnesol production by *C. albicans*, and that these differences could be disguised or overcome by including four trace metals [1 mg/ L Cu(II), Zn(II), Mn(II), and Fe(II)] in the RPMI (2). We prepared a series of RPMI growth media with and without the Acros and Sigma MOPS as well as with and without trace metals (mRPMI and RPMI). After 16 h of shaking at 30°C, 10 flasks without added minerals achieved an average cell density of OD_600_ = 0.90, while 4 flasks with added minerals achieved an OD_600_ = 7.1 (2).

The present paper confirms the relationship between MOPS and trace metal contamination, and then identifies the relative contributions of Cu(II), Zn(II), Mn(II), and Fe(II) to cell growth, farnesol production, and aromatic fusel alcohol production. With regard to cell growth, we compared RPMI with Sigma or Acros MOPS (Table 1) versus mRPMI in triplicate; they differed significantly in their growth rates and cell yields (Fig. 1A). Doubling times for the Sigma MOPS, Acros MOPS, and mRPMI over the 6-to-12-h growth period (highlighted by the dashed box in Fig. 1A) were 2.5 (*R*^2^ = 0.9987), 1.9 (*R*^2^ = 0.9784), and 1.4 (*R*^2^ = 0.9857) h, respectively, and their cell yields after 24, 48, and 72 h were all in the ratio of ca. 1:2:4 (Fig. 1A). These results suggested that the Acros MOPS contained a growth-promoting contaminant which was absent from the Sigma MOPS. However, before pursuing this idea further, we needed to rule out the possibility that the Sigma MOPS contained a growth-inhibiting contaminant. Yeast nitrogen base (YNB) is an optimized defined medium for yeast growth which contains added Fe(II), Zn(II), Cu(II), and Mn(II). Thus, these minerals are absent from RPMI but present in YNB. Triplicate YNB cultures started at an OD_600_ = 0.2 with and without Sigma MOPS grew to the same cell density at 24 h (OD_600_ = 7–8), allowing us to conclude that the Acros MOPS contained a growth-promoting contaminant.

**TABLE 1:** MOPS compounds used in this study

Label, purity	Manufacturer	Lot/Batch #	Date obtained
MOPS, 99%	Acros Organics (17263-1000)	B0131473	06/2011
MOPS, ≥99.5%	Sigma-Aldrich, Inc. (M183-100)	067K01411	01/2012
MOPS sodium salt, ≥99.5%	Sigma-Aldrich, Inc. (M9381-250)	SLCF1714	05/2022

### Influence of MOPS on farnesol production—a subtle artifact

The experiment shown in Fig. 1B had been intended as part of our farnesol assay paper (2). It depicts the per cell whole culture farnesol production for three biological replicates of *C. albicans* grown in RPMI/MOPS at 30°C for 72 h. The precision and reproducibility of this method were shown by the very small variance exhibited among biological triplicates (17 times over 3 days) for YPD and mRPMI grown cultures (2). However, for RPMI/MOPS (Fig. 1B), the first two biological replicates containing Acros MOPS (Fig. 1B, circles) differed dramatically from the third biological replicate containing Sigma MOPS (Fig. 1B, squares). The third replicate grew to a lesser cell density and gave roughly four times more farnesol on a per cell basis than the first two replicates (Fig. 1B). With all three growth curves, there is an *n* = 51, and the average ± SD WPS RE for farnesol is 0.08 ± 0.08; suggesting that the technical variance is small and thus the differences observed are biological. After considerable thought, we attributed these differences to having finished our bottle of MOPS (Acros) after the second replicate and then switched to a MOPS (Sigma) from a neighboring laboratory for the third replicate. For completeness and direct comparison, farnesol production in mRPMI as previously published (2) has been included (Fig. 1B; triangles).

### Trace metal contamination of MOPS

Because of the conspicuous absence of trace metals in RPMI, we hypothesized that these metals were likely the growth-promoting contaminants in MOPS. Thus, we collected seven batches of MOPS from four suppliers from neighboring laboratories and analyzed their trace metal contents by ICP-MS, all without replicates. Significant differences were observed for Cu(II), Zn(II), Mn(II), Fe(II), Ni(II), and Co(II). The differences in Ni(II) and Co(II) were ignored because *C. albicans*, like other yeasts, do not have any Ni(II) or Co(II) requiring enzymes or transport systems (4, 5). In particular, two of the MOPS batches contained high levels of Mn(II), while the other five batches contained none. Neither of the two HEPES batches tested contained Mn(II). One of the two high Mn(II) batches (Fisher Scientific, BP308-100 Lot# 076844) was only 97% pure MOPS, while the other was the Acros MOPS used in Fig. 1. We decided that our triplicate comparisons should only be among batches with ≥99% purity (Table 1): the Acros (circles) and Sigma (squares) MOPS used in Fig. 1, and the currently available MOPS salt used for the remainder of this study in mRPMI (triangles). Growth and farnesol production with the currently available MOPS salt, but without further added minerals, duplicated those observed with the Sigma MOPS used in Fig. 1 (data not shown).

**Fig 2:**
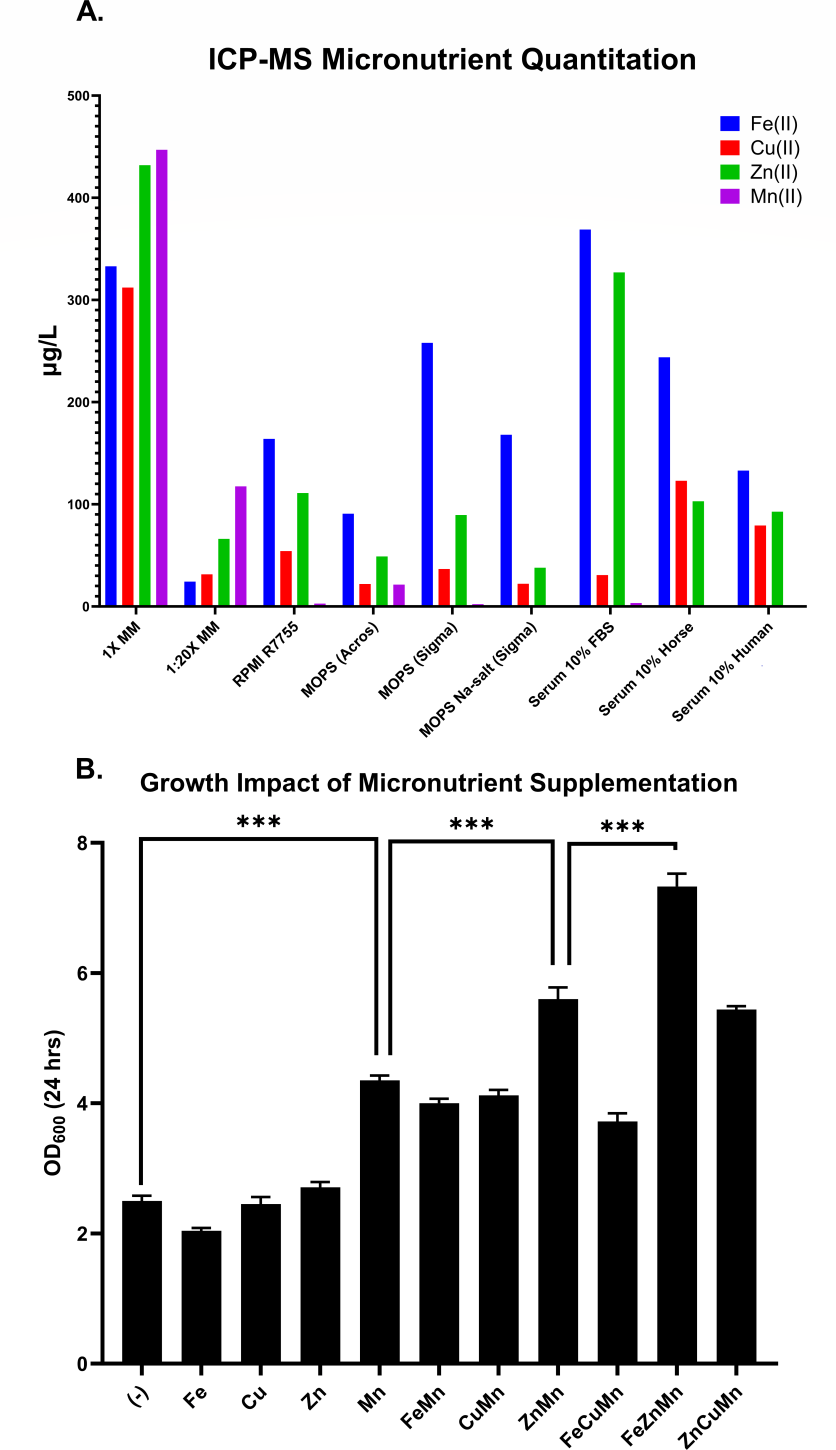
ICP-MS micronutrient quantification and the impact of micronutrients on cell growth. (**A**) ICP-MS analysis of Fe(II), Cu(II), Zn(II), and Mn(II). MM = mineral mix. RPMI R7755 is the basal medium. MOPS (Acros), MOPS (Sigma), and the MOPS salt are described in Table 1. FBS = fetal bovine serum. (**B**) Growth of *C. albicans* in RPMI-1640 with Sigma MOPS salt and individual metals or combination of metals. Each of the indicated metals was at a final concentration of 1 mg/L. Negative (−) is without any mineral addition. The only mineral that enhanced growth by itself was Mn(II). ***P < 0.001 compares: (−) with Mn(II); Mn(II) with Zn(II) + Mn(II); and Zn(II) + Mn(II) with Fe(II) + Zn(II) + Mn(II), highlighting the increase in growth observed with addition of Mn(II), Zn(II), and Fe(II), respectively.

Figure 2A depicts the ICP-MS analysis of Fe(II), Cu(II), Zn(II), and Mn(II) of the 1× mineral mix (MM), the 1:20 diluted mineral mix, commercial RPMI-1640 (Sigma R7755), the three selected MOPS (Table 1), and 10% horse, human, and fetal bovine serum. The serum measurements were included because RPMI-1640 was developed at Roswell Park Memorial Institute (6) to support lymphoblastoid cells in suspension culture and thus was originally intended to be supplemented with serum. The 1× MM data were obtained in duplicate and the relative percent difference (RPD) between technical runs for Fe(II), Zn(II), Cu(II), and Mn(II) were 2.37, 1.45, 1.62, and 1.82, respectively. The RPD of the indium internal standard was 1.26. Significantly, Mn(II) was present in the Acros MOPS but absent in both Sigma MOPS and Sigma MOPS Na-salt, while the other three metals were present at roughly equivalent levels in all three MOPS (Fig. 2A). These data suggest that Mn(II) may be responsible for the growth differences observed with and without mineral mix or using MOPS from different batches or sources (Fig. 1).

### Manganese, zinc, and iron promote optimal growth in RPMI

So far, our results have been directed toward identifying a subtle artifact in the form of metal-contaminated MOPS. We now shift focus to identifying the physiological functions of individual metal supplements. The superior growth exhibited by *C. albicans* in mRPMI could be due to one or more of the four minerals in our mineral mix. To identify the component(s) responsible, RPMI with Sigma MOPS Na-salt was supplemented with Fe(II), Cu(II), Zn(II), and Mn(II), both individually and in combination (Fig. 2B). Mn(II) was the only mineral which increased growth individually; it led to a twofold increase in cell yield (Fig. 2B). The Mn(II) containing medium was then singly spiked with either Fe(II), Cu(II), or Zn(II). The Mn(II) and Zn(II) containing culture showed a threefold increase in cell yield compared to the RPMI control, while the addition of Fe(II) or Cu(II) had no effect (Fig. 2B). Finally, the Mn(II) and Zn(II) containing medium was further spiked with either Fe(II) or Cu(II), showing that the Mn(II), Zn(II), and Fe(II) containing culture gave a cumulative fourfold increase in cell yield compared to the control (Fig. 2B). At no point did the addition of Cu(II) increase growth and at these concentrations the added Cu(II) has no deleterious effects. This latter observation is to be expected because *C. albicans* tolerates up to 20 mM Cu(II) in minimal, defined growth media and up to 50 mM Cu(II) in rich growth media (7). In conclusion, under these conditions, the growth-limiting minerals were Mn(II), followed by Zn(II) and then Fe(II).

### Increased farnesol and aromatic fusel alcohols with added zinc

**Fig 3:**
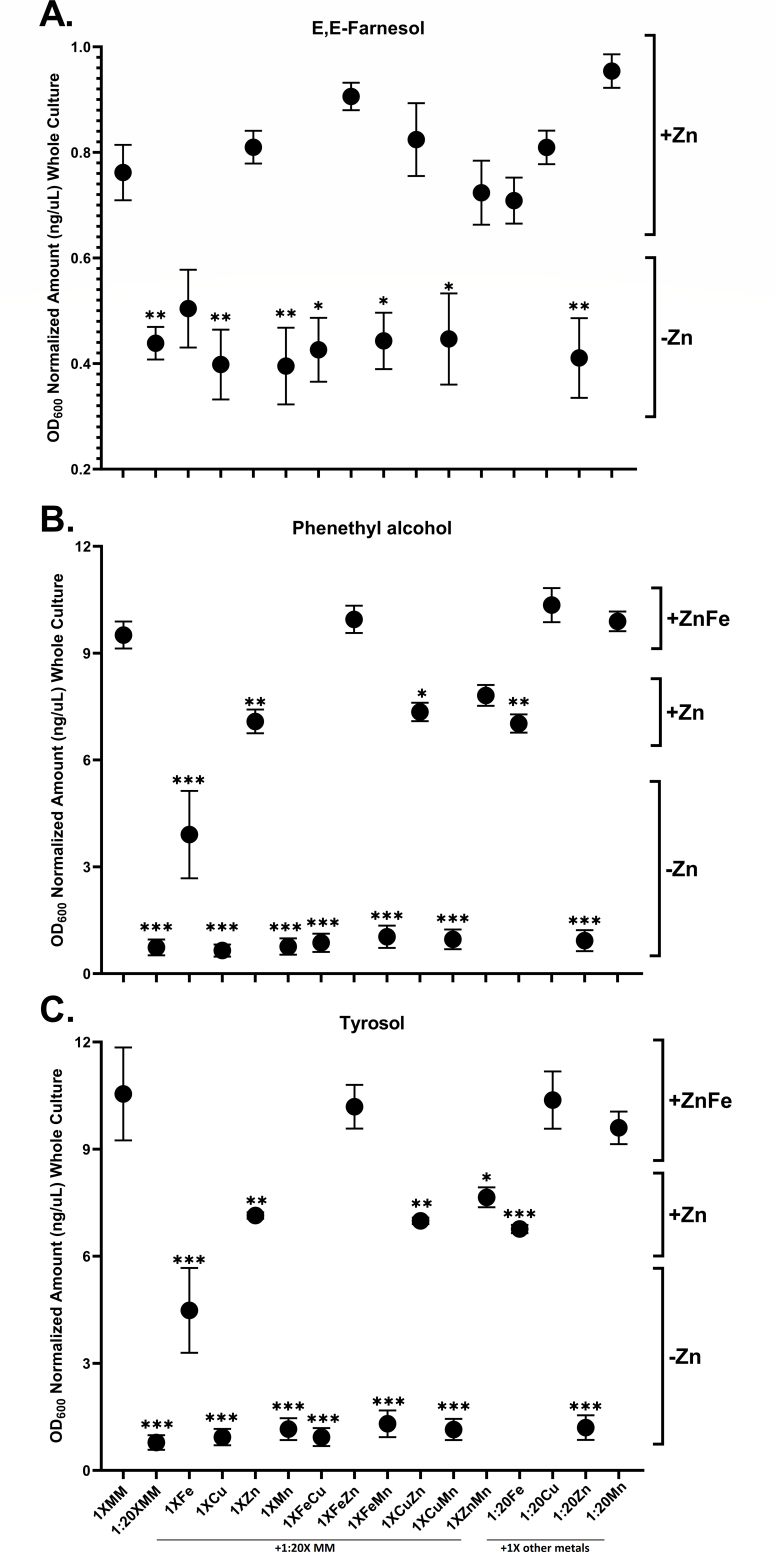
Whole culture GC-FID analyte quantitation. Analyte production normalized to cell density as previously described (2), for RPMI with 16 different metal supplements. MM = mineral mix. 1× MM (higher), and 1:20× MM (lower). Followed by 10 combinations of 1:20× MM supplemented with one or two metals at the higher concentration, and four combinations where all but one of the metals had been raised to the higher level. All combinations were done in biological triplicate with a total of *n* = 48. *X*-axis labels apply vertically to all graphs. *P  <  0.05, **P  <  0.01, and ***P  <  0.001 are all compared to 1× MM cultures. (**A**) E,E-farnesol production showing a bimodal distribution; all cultures with lower levels of Zn(II) had significantly lower amounts of farnesol produced when compared to cultures with the higher Zn(II) levels. (**B**) Phenethyl alcohol production followed a trimodal distribution with an initial increase with higher Zn(II) and a further increase with higher Fe(II). (**C**) Tyrosol production also followed a trimodal distribution.

To assess the effects of added minerals on farnesol and aromatic fusel alcohol production, we first established the lowest mineral concentrations sufficient for close to optimal growth. This precaution was taken to ensure the differences observed were directly due to mineral abundance rather than indirectly due to their effects on growth. Triplicate RPMI-1640 cultures supplemented with varying levels of mineral mix (MM) were grown 24 h. The cultures grew to average OD_600_ values of 7.9 ± 1.8 (4×), 7.6 ± 1.7 (1×), 7.9 ± 1.6 (1:10×), 7.2 ± 0.9 (1:20×), 3.3 ± 1.6 (1:50×), and 2.1 ± 0.3 (1:100×). Therefore, RPMI with 1:20 X MM was used in all subsequent experiments assessing production levels for farnesol (Fig. 3A) and the aromatic fusel alcohols (Fig. 3B and C).

In addition to the 1× and 1:20× controls, we had 10 cultures where the 1:20× mineral mix was supplemented with one or two minerals at full (1×) strength, and another four cultures where three of the metals were at full strength while the fourth remained at 1:20×. All the cultures were in biological triplicate (Fig. 3). Farnesol production followed a bimodal distribution in that cultures with added Zn(II) produced roughly two times the farnesol as those without added Zn(II) (Fig. 3A). Similarly, phenethyl alcohol and tyrosol production followed a trimodal distribution in that cultures with added Fe(II) and Zn(II) mimicked the 1× MM (mRPMI) in having roughly 10-fold higher production than the 1:20× MM control, while those with added Zn(II) only were roughly 6-fold higher (Fig. 3B and C). Having almost identical results for phenethyl alcohol (Fig. 3B) and tyrosol (Fig. 3C) would be consistent with their production by either the Ehrlich pathway (8) or the aromatic amino acid biosynthetic pathway (9). This identity between the two fusel alcohols should not be expected in other growth media.

### Antifungal susceptibility testing

Given the above-described variations in cell growth and farnesol production, to what extent should we worry about the accuracy of antibiotic testing protocols? As a partial answer, we conducted two RPMI-based screens with and without mineral mix. The first was the automated VITEK 2 system which tested *C. albicans*, *C. krusei*, and *C. parapsilosis* versus fluconazole, voriconazole, caspofungin, and micafungin. The results with and without the added mineral mix were identical in all cases (all sensitive) and in each case the minimum inhibitory concentration (MIC) value remained unchanged. However, for the VITEK 2 system, uncharacterized MOPS was always included as part of the lyophilized nutrients provided by the supplier and for that reason we also performed fluconazole sensitivity testing in 96-well plates in biological triplicate, using 0 to 64 µg/mL fluconazole in RPMI buffered with MOPS salt with and without added 1× mineral mix following the CLSI and EUCAST protocols (3). The results were equivalent in all cases; the cells were classified as sensitive with MIC values following the CLSI protocol of 0.149 ± 0.013, and 0.154 ± 0.001 with and without 1× MM and following the EUCAST protocol of 0.158 ± 0.018 and 0.172 ± 0.021 with and without 1× MM (Table 2).

**TABLE 2:** Fluconazole CLSI and EUCAST AFST MICs (µg/mL) of *Candida albicans* (SC5314) in RPMI/MOPS Na-salt ±1× MM. Examined in triplicate wells in 96-well plate and reported as mean ± SD

1× MM	(−)	(+)
CLSI	0.154 ± 0.001	0.149 ± 0.013
EUCAST	0.172 ± 0.021	0.158 ± 0.018

## DISCUSSION

Transition metals Fe(II), Cu(II), Zn(II), and Mn(II) are essential for life and therefore it is not surprising that fungal pathogenicity, virulence, host inflammatory induction, immune evasion, and drug resistance are intimately linked with the bioavailability of transition metals (10–13). These transition metals play a vital role in a multitude of processes as cofactors in many proteins involved in the antioxidant response (CuZnSOD), mitochondrial metabolism (Fe-S clusters), and gene expression, among others (10, 14). Studies dealing with microbe-metal interactions generally examine a microbe’s ability to achieve homeostasis, where low levels of the metal are required but higher levels can be deleterious. For instance, micromolar (µM) levels of Zn(II) are usually sufficient for cell growth (15, 16), whereas millimolar (mM) levels of Zn(II) can shift dimorphic fungi from growing as mycelia to yeasts. This phenomenon has been observed for *C. albicans* (17–19), *Histoplasma capsulatum* (20), *Mucor rouxii* (21), and *Ceratocystis ulmi* (22). The present paper investigates the consequences of transition metals being absent from the commonly used growth medium RPMI-1640. For *C. albicans*, we observed dramatic differences in growth rates and cell yields as well as in the per cell production of farnesol and aromatic fusel alcohols. There are four take-home messages from this work. First, RPMI does not contain any added trace metals. Second, there is batch-to-batch variance in mineral contamination of MOPS, the most commonly used buffer for RPMI-1640. Thus, literature comparisons of the relative merits of different buffers, for example, MOPS versus HEPES, should consider the type and extent of trace metal contamination of the respective buffers (23, 24). Third, in addition to the rate and extent of cell growth, these trace metal variations may cause biological variance in a great many ways, as we observed for farnesol and aromatic fusel alcohol production. And fourth, researchers may wish to supplement commercial RPMI-1640 with added minerals. We now provide 1 mg/L (ca. 4 µM) of Cu(II), Zn(II), Mn(II), and Fe(II) which we refer to as mRPMI (2). Please note that this mineral mix has not been optimized. It is the same one that we used in a chemically defined glucose-phosphate-proline (GPP) medium for the growth of *C. ulmi* (25), which in turn was based on a defined medium for the growth and sporulation of the bacterium *Bacillus cereus* (26).

Given the many differences between RPMI- and mRPMI-grown cells, should we question the validity of published antibiotic resistance studies conducted with RPMI-MOPS grown cells? At present we feel that caution is warranted, but nothing beyond caution. In this paper, a standardized antifungal testing system for *C. albicans* versus four common antifungals gave identical results with and without an added mineral mix. Also, from a more general perspective, unsupplemented RPMI contains sufficient trace metals (Fig. 2) for cell growth to an OD_600_ of 1–2 after 24 h; the added mineral mix is needed to achieve OD_600_ values of 6–10. Low levels of divalent cations such as Fe(II), Cu(II), and Zn(II) are commonly complexed to amino acids on their free carboxyl groups, or alternatively they can be leached from glassware (16). Thus, unsupplemented RPMI should still be suitable for studies on germ tube formation, biofilm formation, antibiotic resistance, or any other phenomenon requiring only a limited number of cell doublings. However, it is still possible that these trace metal variations could alter farnesol production (Fig. 1B) and thus indirectly influence: (i) whether an antibiotic is fungistatic or fungicidal, (ii) whether the antibiotic exhibits a trailing end point phenomenon, (iii) polymicrobial growth between a fungus grown in RPMI and a bacterium grown in, for instance, brain-heart-infusion agar, (iv) the effects of added serum or, (v) metal activated transport systems which might be relevant for the import or export of a particular antibiotic (27). Finally, as a general theme, whenever *C. albicans* becomes metal ion deficient and shifts from respiration to fermentation, it is well established that anaerobically grown cells may be more antibiotic-resistant than aerobically grown cells (28).

When evaluating the influence of trace metals on fungi, one also must consider the nutrient conditions under which the inocula had been grown. Two Zn(II)-related examples are relevant to the RPMI/mRPMI distinction. The first concerns findings from the Soll laboratory (16, 29) that in a chemically defined growth medium (30) at 25°C, *C. albicans* 3153A with ≤1 µM Zn(II) will enter stationary phase because of Zn(II) starvation. This feature is often advantageous because all the stationary phase cells are singlet, unbudded cells that are now totipotent. That means when diluted into fresh nutrient media at 37°C, they can resume growth as either synchronized mycelia or buds (16, 29). In contrast, with excess Zn(II) (9 µM) more cells were produced, but the stationary phase cells were a heterogenous mixture of budded cells. Interestingly, these authors concluded (16) that when Zn(II) was not limiting, it appeared that one or more other divalent cations were limiting. Our evidence in the current paper identifies Mn(II) as the other divalent cation.

The second concerns polyphosphate as an anionic polymer acting as a divalent cation sequestering and storage agent. Like many microorganisms, when presented with an excess of phosphate *Candida* sp. responds by synthesizing polyphosphate and storing it in their vacuoles (31). This enhanced level of accumulated Zn(II) is not inhibitory to growth (32) and because Zn(II) is localized in the vacuole, it explains why Zn(II) accumulation is unidirectional (32, 33) and not removed by repeated washing. Significantly, the cellular content of polyphosphate is elevated in Zn(II)-deficient (19, 32) and potassium-deficient yeasts (34).

We found that Zn(II) was the most important metal for stimulating the production of farnesol (Fig. 3A) and the aromatic fusel alcohols (Fig. 3B and C). It was the only metal that stimulated farnesol production by itself and in the MOPS comparisons, the Acros MOPS contained threefold less Zn(II) and produced three- to fourfold less farnesol (Fig. 1B). Zn(II) is an essential micronutrient for which a precise homeostasis is needed for survival. Up to 9% of a eukaryotic proteome requires Zn(II), and these proteins can be subdivided into those which are enzymes (47%), transcription factors (44%), storage and transport (5%), and cell signaling (3%) components (35, 36). Given this extensive use of Zn(II), it is not surprising that altered levels of Zn(II) can influence fungal growth in many ways. For *C. albicans*, *C. dubliniensis*, and *C. tropicalis*, the limitation for Zn(II) specifically resulted in formation of large, spherical yeasts termed Goliath cells (37). Thus, there is a plethora of individual targets, both direct and indirect, for this Zn(II) effect. The likelihood of an indirect mechanism is based on the highly branched sterol biosynthetic pathway wherein carbon flow through FPP can be directed towards farnesol, geranylgeraniol, dolichol, and ubiquinones as well as to sterols (1). It is well established that antibiotics such as fluconazole, zaragozic acid, and terbinafine, which block sterol biosynthesis also increase farnesol production (1), presumably by elevating the FPP pool size. Thus, any metal-directed treatment that limited sterol biosynthesis could also elevate the FPP pool size and farnesol production.

### The importance of manganese [Mn(II)]

Our experiments showed that Mn(II) was most important for stimulating cell growth. It was the only metal that increased growth by itself (Fig. 2B) and in MOPS comparisons, the Acros MOPS contained 9.6-fold more Mn(II) and achieved ca. 3-fold more growth. This finding is not new. Lilly and Bartnett (38) noted that the omission of Mn(II) resulted in decreased cell yields. They said that fungal inocula usually carried sufficient Mn(II) for growth in the first two passages; no growth only resulted from the third passage in the absence of added Mn(II). These observations were consistent with studies on Mn(II) transport into non-exchangeable pools within yeast cells (39), presumably into polyphosphate-containing vacuoles. Furthermore, the analysis of Mn effects in fungi at that time was heavily dependent on the brand of MgSO_4_ chosen because several were heavily contaminated with Mn(II) (38). In this regard, Fig. 2A shows that commercial RPMI, most brands of MOPS, and most sera are distinctly Mn-deficient. Thus, decisions regarding whether media should be supplemented with Mn(II) are dependent on known physiological functions of Mn(II). This field has recently had a resurgence of interest (40–44), including reports on Mn(II) being required for microbial pathogenesis (40), promoting fungal lifespan (41), and maintaining Mn(II) homeostasis and normal hyphal development and conidiation (42). The mechanisms of Mn(II) based nutritional immunity versus *C. albicans* were recently explored by Wildeman et al. (43). They identified two divalent metal transporters in *C. albicans* Smf12 (19.2270) and Smf13 (19.5022) required for cellular Mn(II) accumulation. The *smf12Δ/Δ* and *smf13Δ/Δ* mutations had 10- to 80-fold less Mn(II), exhibited impaired activity of mitochondrial Mn-superoxide dismutase 2 and cytosolic Mn-superoxide dismutase 3, accompanied by dramatic loss in cell surface phosphomannans and glycosylation of proteins. The mutants were also defective in forming hyphal filaments and displayed a significant loss in virulence (43). These deficiencies were rescued by supplementation with 10 µM Mn(II), but not by supplementation with the same levels of Fe(II), Cu(II), or Zn(II) (43). Coremia formation in *Penicillium claviforme* and *Penicillium clavigerum* is also Mn(II) dependent. In Mn(II) free media the fungi grew but did not produce coremia (45). Finally, kidneys are the primary target for candidiasis in disseminated mouse models and Wildeman et al. (43) noted up to a 40% loss of whole kidney Mn(II) levels in both male and female mice 72 h after injection by wild-type *C. albicans*. However, despite these findings on the overall importance of Mn(II), there are comparatively few individual targets requiring Mn(II). Chief among these are superoxide dismutase, RNA polymerase (46), and the Golgi-bound mannosyl-transferases required for phosphomannan cell wall synthesis and other aspects of cell wall integrity (40, 47). Further reasons for providing fungal cells with optimal concentrations of Mn(II) include: (i) Mn(II) is a physiologically relevant activator of TOR in yeasts (48); (ii) Mn(II) transporters are widespread in fungi (49); (iii) Mn(II) limitation triggers the unfolded protein response (44); (iv) the toxin candidalysin is upregulated by Mn(II) limitation (44); (v) wild-type *C. albicans* grown in Mn(II)-starved media were moderately more sensitive to fluconazole and miconazole but *smf12Δ/Δ* mutants were hypersensitive to both (44); and (vi) 90 min following Mn(II) starvation the *C. albicans* transcriptome was markedly similar to that of cells that were Hsp90 inhibited, either genetically or pharmacologically by geldanamycin (44).

Finally, microbial pathogens encounter nutritional immunity whereby infected host tissues sequester essential nutrients such as iron (50) and other trace metals (51) to prevent or retard microbial growth. Efforts to stimulate these metal-starved conditions *in vitro* are more complicated because the nutritional immunity employed may be niche-specific (51). For instance, *C. albicans* is subject to iron and zinc nutritional immunity during disseminated infection but not to iron nutritional immunity during oral pharyngeal infection (51). Of particular relevance to the RPMI-1640 story, both bacteria (52) and fungi (40) battle the mammalian immune system for Mn(II), where the dominant mechanism to sequester essential Mn(II) and Zn(II) is via the metal binding protein calprotectin (40, 43, 53). Amazingly, calprotectin can constitute 40–50% of the cytoplasmic protein content of neutrophils (54, 55). Calprotectin is a potent antifungal versus *C. albicans* (56) and can reach concentrations exceeding 1 mg/mL in infected tissues (57). Perhaps its presence in such high amounts in neutrophils explains why neutrophils are candicidal while macrophages are not (58). In sum, the precise mechanism by which Mn(II) stimulates growth is still elusive and intriguing.

## MATERIALS AND METHODS

### Organism, growth media, and growth conditions

The strain used throughout, for both growth curves and mineral assessment, was *C. albicans* SC5314 (59). Media used were RPMI-1640 (R7755 Millipore-Sigma, St. Louis, MO, USA) to which we added the recommended levels of glucose, l-glutamine, and MOPS (3-(N-morpholino)propanesulfonic acid) (60). Details of the three MOPS used are in Table 1. The RPMI containing the Sigma MOPS sodium salt and 1× mineral mix is referred to as mRPMI. Growth curves were performed in Fernbach flasks as previously described (2). Our 1× mineral mix provided a working calculated concentration of 1 mg/L of Cu(II), Zn(II), Mn(II), and Fe(II). AFST assessment utilizing the VITEK2 system was performed with quality control strains *C. albicans* ATCC 14053, *Candida krusei* ATCC 6258, and *Candida parapsilosis* ATCC 22019.

Experiments defining alternate mineral components and those establishing upper and lower mineral concentrations for optimal growth were performed in 3 mL of culture media using independent SC5314 isolates. Inocula were grown overnight in mRPMI and washed with deionized water to remove excess minerals. Cultures were inoculated at an OD_600_ of 0.2 and shaken at 225 rpm at 30°C. Cell growth was observed via OD_600_ values assessed using SpectraMax 384 Plus (Molecular Devices, San Jose, CA, USA). Analyte assessment of upper and lower mineral levels used 60 mL cultures in 250 mL flasks after 24 h.

### ICP-MS

Samples were prepared and assessed following Environmental Protection Agency and National Environmental Methods Index guidelines for quality control (61). The RPD between duplicate samples was assessed for data quality. Indium was added and analyzed as a surrogate to measure recovery. The method followed was an American Public Health Association standard method for analysis of digested water samples. Briefly, samples and standards were digested using concentrated nitric acid and hydrogen peroxide on a MARS Xpress microwave digestion system. The digests were diluted to 1% nitric acid prior to analysis on Thermos ICAP-RQ inductively coupled plasma mass spectrometer.

### Analyte quantitation

Farnesol and the fusel alcohols were assessed as previously described (2). Briefly, 10 mL of either whole culture, fractionated pellet (reconstituted in 10 mL fresh media), or supernatant were extracted into 1.5 mL ethyl acetate with 17.2 ng/µL 1-tetradecanol as internal standard. For each sample, 1 mL of 5 M NaCl was added to assist with phase separation. Extracts were analyzed on an Agilent Technologies 7890 B gas chromatograph (Santa Clara, CA, USA) equipped with autosampler and flame ionization detector (FID). Samples (2 µL) were injected onto HP-Innowax (Agilent 19091N-1331) column with hydrogen as carrier gas at an initial temperature of 90°C ramped at a rate of 30°C/min to a final temperature of 245°C and held there for 7 min. Data files were batched using OpenLab Services (Agilent Technologies) with software calculated response factors based on standard calibrator solutions. Proper control samples were run and batched with each biological triplicate experiment. Data quality was assessed using the relative error between the whole culture and the sum of pellet and supernatant values (WPS RE).

### Broth dilution antifungal susceptibility testing

We assessed the influence of added minerals on the reliability of standardized AFST following both the European Committee on Antimicrobial Susceptibility Testing (EUCAST) and the Clinical Laboratory Standards Institute (CLSI) guidelines (3). We used the VITEK2 system (bioMérieux) following the protocols of Wiederhold on *C. albicans* versus fluconazole, voriconazole, caspofungin, and micafungin utilizing *C. albicans* ATCC 14053, *C. krusei* ATCC 6258, and *C. parapsilosis* ATCC 22019 as control strains. In this automated system, nutrients (RPMI, glucose, and MOPS) and antifungals are added in lyophilized cassettes, permitting addition of mineral mix as part of the yeast suspensions being tested.

### Statistical analysis

Statistical analyses were performed using Graphpad Prism Software (Version 10.2.2, San Diego, CA, USA) and Microsoft Excel (Version 16.85, Microsoft Office, Las Vegas, NV, USA). Data are represented as the mean ± SD of three biological replicates. Differences between MOPS batches were assessed by one-way ANOVA with Dunnett multiple comparisons test. The differences were considered significant at *P* < 0.05 (*P < 0.05, **P < 0.01, and ***P < 0.001).
